# Operation

**Published:** 1888-09-08

**Authors:** William Ewart Henley

**Affiliations:** in "A Book of Verses."


### OPERATION.

You are carried in a basket,
Like a carcase from the shambles,
To the theatre, a cockpit,
Where they stretch you on a table.

Then they bid you close your eyelids,
And they mask you with a napkin,
And the anæsthetic reaches
Hot and subtle through your being.

And you gasp, and reel, and shudder
In a rushing, swaying rapture,
While the voices at your elbow
Fade—receding—fainter—farther.

Lights about you shower and tumble,
And your blood seems crystallising—
Edged and vibrant, yet within you
Racked and hurried back and forward.

Then the lights grow fast and furious,
And you hear a noise of water,
And you wrestle, blind and dizzy,
In an agony of effort.

Till a sudden lull accepts you,
And you sound an utter darkness......
And awaken......with a struggle .....
On a hushed, attentive audience.

—*William Ewart Henley*, in "A Book of Verses."

